# The measurement, sources of variation, and factors influencing the coupled and coordinated development of rural revitalization and digital economy in China

**DOI:** 10.1371/journal.pone.0277910

**Published:** 2022-11-28

**Authors:** Meng Du, Yanshun Huang, Hai Dong, Xiangjun Zhou, Yipan Wang

**Affiliations:** 1 Department of Finance, Shandong Technology and Business University, Yantai, Shandong, China; 2 Collaborative Innovation Center for Financial Service Transformation and Upgrading, Shandong Technology and Business University, Yantai, China; 3 School of Land Science and Technology, China University of Geosciences (Beijing), Beijing, China; 4 School of Investment and Construction Management, Dongbei University of Finance and Economics, Dalian, Liaoning, China; 5 School of Business, Wenzhou University, Wenzhou, Zhejiang, China; Szechenyi Istvan University: Szechenyi Istvan Egyetem, HUNGARY

## Abstract

An evaluation index system for the coupled and coordinated development of China’s digital economy and rural revitalization, including a total of 46 indicators for the digital economy and rural revitalization subsystems, was constructed and combined with the entropy weight method, the coupling coordination degree model, Zhou’s constraint identification index, the Dagum Gini coefficient decomposition method, and the panel spatial econometric model to analyze the level of coupled and coordinated development of China’s digital economy and rural revitalization. The results found that: (1) the coupling and coordination between the two have gradually improved. The constraints of the digital economy on rural revitalization were gradually alleviated from 2011 to 2015, but after the 19th Party Congress, the development trend of rural revitalization has significantly outstripped the digital economy. (2) the spatial differences in the degree of coupling and coordination between the two are dominated by inter-regional differences and show significant spatial convergence and spatial correlation. Differentiated digital economy development strategies and more radiation in polarized areas are important for reducing regional differences in the level of coupling and coordination between the digital economy and rural revitalization. This will help China’s digital countryside grow more efficiently.

## 1. Introduction

The digital economy that empowers rural development is essential in constructing digital China, rural revitalization, and sustainable development strategies. The report of the 19th National Congress of the Communist Party of China (CPC) innovatively proposed the rural revitalization strategy of "prosperous industry, pleasant ecology, civilized countryside, effective governance, and prosperous living." Since then, the government has introduced a series of policies to promote the digital economy to empower rural revitalization. The Chinese central government’s Document No. 1 proposed the concept of digital countryside development in 2018. The Government Work Report also proposed promoting "Internet + rural areas," improving rural circulation networks and supporting the growth of rural e-commerce and rural express in 2019. The Digital Agriculture and Rural Development Plan emphasizes the deep integration of digital technology into the agricultural and rural economies in 2020. The Outline of the 14th Five-Year Plan for the "National Economic and Social Development of the People’s Republic of China" and Vision 2035 emphasize the need to accelerate the construction of the digital countryside, build an integrated information service system for agriculture and rural areas, establish a mechanism for providing universal access to agriculture-related information, and promote the digitization of rural management services. All these policies have clearly defined the strategic direction of the digital economy to drive the modernization of agriculture and rural areas, and the digital economy has become a vital force to support the development of rural revitalization. With the comprehensive coverage and convenience of the Internet, the digital economy can effectively solve the "last kilometer" of rural construction and become an effective engine and sustainable driving force for the revitalization of the countryside. At the same time, the revitalization of the countryside provides good fertile ground for the innovative development of the digital economy. There is a lot of room in the countryside for digital governance, industrial digitization, digital infrastructure construction, and digital industrialization, which is important for China’s digital economy to grow and improve its quality and efficiency.

Academics have actively explored the relationship between the digital economy and rural revitalization. A series of studies have been conducted from the perspectives of agricultural supply-side reform, digital finance, rural e-commerce, rural governance, ecological livability, rural civilization, etc. The research results mainly focus on the positive impact of the digital economy on rural revitalization, which is reflected in the following aspects: First, the digital economy improves residents’ incomes, promotes household consumption, enhances the efficiency of poverty alleviation, and improves entrepreneurial vitality [1–9]. Secondly, digital technology can accurately obtain agricultural production data, promote precise control of agriculture, and realize the digitization of agricultural production and agricultural operations. The digital economy also enhances the voice of farmers in the agricultural industry chain. At the same time, digital finance improves the possibility of small farmers’ access to financial services and provides strong support for the integrated development of the three rural industries [10–12]. Thirdly, it realizes the transparency of the agricultural production process, forcing farmers to adopt green production techniques and guarantee the quality and safety of green agricultural products. The digital monitoring platform achieves the wisdom of rural environmental protection and enhances the ecological digital level [10, 13–16]. Fourth, digitalization promotes the construction of rural civilization, with digital technology embedded in rural public spaces and public facilities, forming new industries such as smart, field complexes, and characteristic towns [17–20]. Furthermore, the digital economy raises the level of rural human capital, enhances farmers’ market access, and provides entrepreneurial employment opportunities [21–24]. Fifth, the digital economy has broken the original social, relational, and geopolitical structures, reshaped the rural governance pattern, expanded new ways and means for pluralistic participation in rural governance, and realized the digitalization of governance [16, 25–28]. Sixth, the digital economy has broadened students’ access to knowledge through digital technology, promoting balanced and equitable education in urban and rural areas [19, 29–31] and, by realizing the downward sharing of high-quality medical resources, promoting balanced urban and rural healthcare [32–34]. The Internet platform provides convenient services, public services, consulting services, and e-commerce to alleviate poverty [35–39].

As can be seen from the above, the existing literature focuses on studying the digital economy and rural revitalization unilaterally, while the study from the perspective of the coupled and coordinated development of the two is relatively blank. As both the digital economy and rural revitalization are comprehensive development indicators, their development is bound to be intertwined and influence each other. However, the degree of coupling and coordination between the digital economy and rural revitalization in China and their mechanisms of action are still unclear, and there is regional heterogeneity, with the development level of the digital economy in some regions seriously lagging, making it challenging to meet the needs of rural revitalization and in urgent need of solid investment. In some regions, the level of digital economy development is over-saturated in terms of the demand for rural revitalization, so government investment in the digital economy in these regions can be appropriately reduced, and the limited funds can be redirected to regions where the digital economy is seriously lagging. Quantifying the degree of coupling between rural revitalization and the digital economy in China can help measure the degree of lagging digital economy in different regions relative to the development needs of rural revitalization, identify bottlenecks in the practice of digital empowerment for rural revitalization, and help optimize government investment in the development of the digital economy in rural areas.

The possible marginal contributions: First, by constructing an indicator evaluation system for the two subsystems of the digital economy and rural revitalization, a coupling coordination degree model is applied to analyze the coupling coordination degree of the two subsystems, revealing the regional gaps and spatial correlations of the coupling coordination between the two. Secondly, the "Zhou’s Constraints Identification Index" is proposed and constructed for the first time to identify and visualize the key elements and critical regions that constrain the coupling and coordination between China’s digital economy and rural revitalization and to provide a reference for the government’s precise investment in rural revitalization. Thirdly, the Dagum Gini coefficient and its decomposition are used to reveal the spatial differences and sources of differences in the coupling and coordination between the digital economy and rural revitalization in China. Fourthly, the absolute and relative spatial beta convergence models are applied to verify the convergence of the coupling and coordination degrees of China’s digital economy and rural revitalization and explore their influencing factors. This paper provides a basis for comprehensively grasping the historical evolution pattern, spatial distribution differences, screening key constraints, and convergence characteristics of the coupling and coordination degree of China’s digital economy and rural revitalization. It also promotes a new pattern of digital rural development.

## 2. Theoretical analysis

### 2.1 Examine the mechanism of interaction between rural revitalization and the digital economy

**Fig 1:**
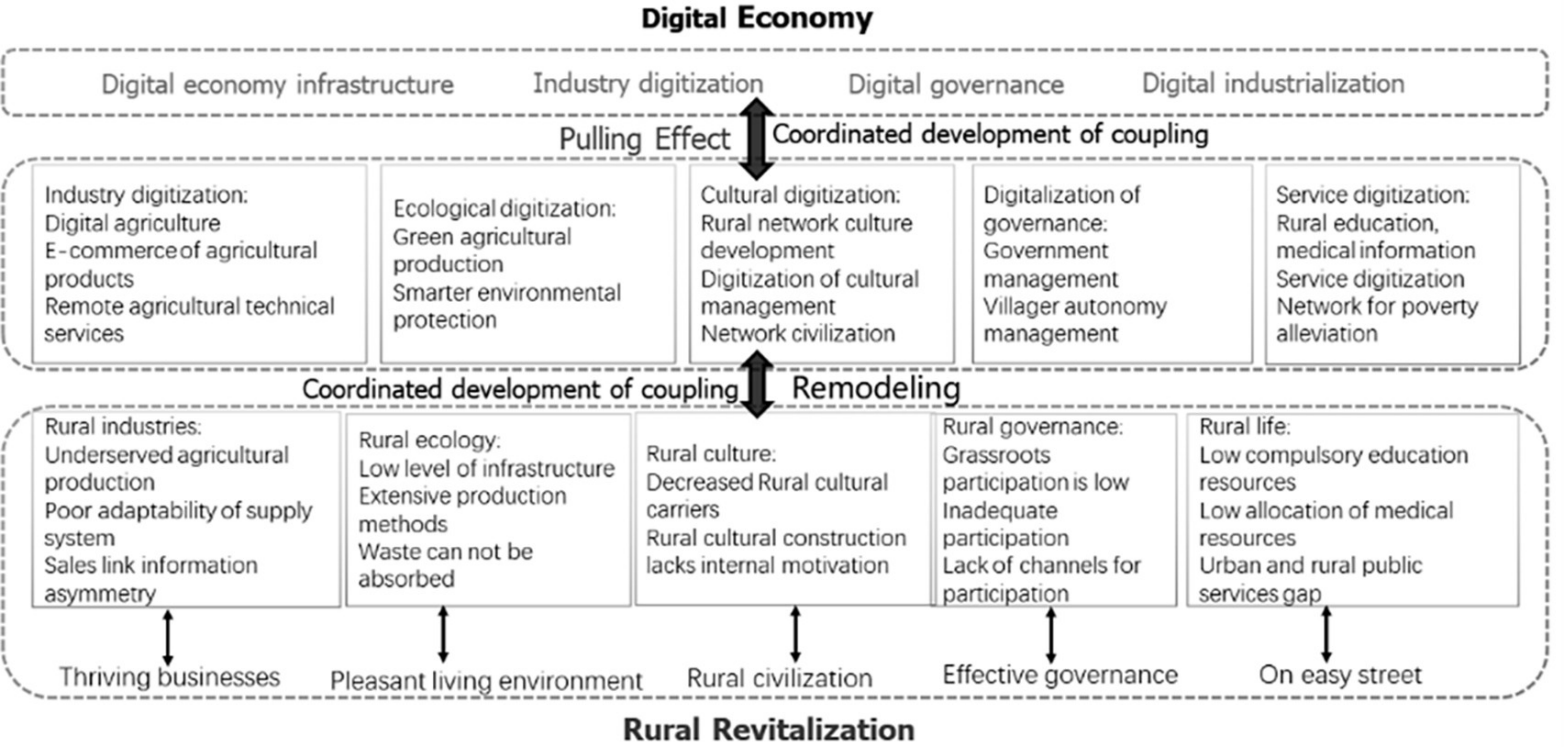
Mechanism of interaction between the digital economy and rural revitalization.

The digital economy and rural revitalization aim to serve disadvantaged groups in rural areas, lift them out of poverty, and increase their incomes, so the two have the same intrinsic goal and must have a coupling interaction. At present, a new generation of digital technology is accelerating its penetration into agriculture and rural areas, providing an excellent opportunity for digital empowerment to promote rural revitalization. The deep integration of digital technology and agricultural and rural development will realize scientific production, visualization of governance, intelligent life, and convenient services in the countryside, promoting prosperous industry, ecological livability, civilized countryside, effective governance, and affluent life in the countryside. At the same time, rural revitalization provides space for the reshaping development of digital economy infrastructure, digital governance level, industry digitization, digital industrialization, etc. The specific path and inner mechanism of the interactive coupling and synergistic development of the digital economy and rural revitalization are shown in Fig 1.

- The interaction mechanism between the digital economy and the prosperity of rural revitalization industries. The digital economy improves the ability to capture information in agricultural production and obtains accurate agricultural production data to achieve precise control of agriculture. With the support of digital technology, the implementation of e-commerce transformation has solved the information blockage existing in the traditional agricultural market. With the openness of the internet platform, barriers to the application and service of agricultural technology are broken, and the technical needs of farmers are quickly delivered. Digital finance achieves the allocation of supply and demand of rural funds at a lower cost, solves the problems of financial geographical discrimination and supply-based financial inhibition in rural areas, improves the possibility for small farmers to access financial services, and provides strong support for the integrated development of the three rural industries. The need for digitalization in rural revitalization drives the development of digital economy infrastructure and industry digitization, which in turn drives the development of digital economy infrastructure.
- The interaction mechanism between the digital economy and ecological livability in rural revitalization. The development based on the Internet of Things realizes the transparency of the agricultural production process and the delicate operation of production factors such as fertilizers, chemical fertilizers, and pesticides to protect the rural ecological environment. With the new generation of IoT and mobile internet platforms, we can strengthen the resource utilization and pollution prevention of livestock and poultry breeding, improve the control of agricultural surface source pollution, and improve the information level of rural ecological and environmental improvement. China’s "beautiful countryside" project will rise in level as a result of improving the living environment in rural areas. This, in turn, will help the country’s digital economy industry and digital economy infrastructure get better and better.
- The interaction mechanism in rural revitalization is between the digital economy and rural civilization. Urbanization has caused an exodus of people from the countryside. "Hollow villages" are emerging, and intangible cultural heritage is gradually disappearing. The digital economy can more economically and efficiently record local cultural characteristics and resources with high humanistic value, such as multimedia. Digital technology is embedded in rural public spaces and facilities, forming new industries such as intelligent rural tourism, field complexes, and characteristic towns, using digital technology to disseminate and display the characteristics of rural culture, enhance the expressiveness and attractiveness of rural culture, and realize rural cultural revitalization. The popularity of the Internet has improved farmers’ access to information and provided a new model and channel for farmers’ training. Cultural digitization furthers the development of digital industrialization in the digital economy.
- Digital inclusion and good governance. Big data and rural governance, rural governance grid, "rural + government services", digital rural and public services, and intelligent rural have become the primary forms of rural governance. Digital technology enables data sharing and sharing to break down information. Institutional barriers broaden the channels for villagers to participate directly or indirectly in governance, transform rural governance’s primary mode from a one-dimensional vertical management mode to a pluralistic shared governance mode, and improve rural governance performance. People in rural areas are getting more and more of their government work done online, which helps the digital economy develop.
- The interaction mechanism between the digital economy and the improvement of people’s livelihoods in rural revitalization. Digital technologies break the physical distance between teachers and students in urban and rural areas, share quality education resources co-efficiently, and promote balanced and fair education in urban and rural areas. The Internet platform is used to share quality medical resources downward, promote balanced urban and rural medical care, and provide convenient, public welfare, consulting, and e-commerce services. E-commerce poverty alleviation encourages people to buy things online and start businesses, which in turn encourages the transformation and upgrading of digital industries in poor areas. It also has a big impact on raising the incomes of poor families and improving their living standards.

To sum up, the digital economy empowers rural revitalization in such aspects as industrial prosperity, ecological dependence on livability, rural civilization, rural governance, and people’s livelihoods, respectively. Rural revitalization, on the other hand, makes room for the development of the digital economy in areas like digital economic infrastructure, industrial digitization, governance digitization, and digital industrialization, and the two work together to help each other grow.

### 2.2 Analysis of the existence of optimal coupling and coordination

From the perspective of development theories, the digital economy can promote capital accumulation through its low cost and comprehensive coverage and change the level of technology to promote rural revitalization. In a complex economic system, the connection between the digital economy and the countryside is complicated, and in some regions, the development even shows deviation. Therefore, it is of solid theoretical value to prove the existence of optimal coupling and coordination from a theoretical perspective, which is conducive to sorting out the coupling mechanism. In this paper, we look at two investment factors: the digital economy and rural revitalization. We assume that labor and technology are not factors in the analysis:

$$Y=D^{\alpha }R^{\beta }{\left(TL\right)}^{\left(1-\alpha -\beta \right)}$$ (1)

Where: economic output *Y* is determined by the digital economy *D*, rural revitalization *R*, technology *T*, and labor force *L*. *α* and *β* are the elasticities of the digital economy and rural revitalization respectively, both taking values greater than 0 and less than 1. For analysis, the growth rate of the labor force is assumed to be *n*,the depreciation rate of the digital economy *δ*_*D*_ and the depreciation rate of rural revitalization *δ*_*R*_ are equal, i.e., *δ*_*D*_ = *δ*_*R*_ = *δ*. The propensity to save in the digital economy and rural revitalization are *S*_*D*_ and *S*_*R*_ respectively. The accumulation equation for the digital economy is *D* = *s*_*D*_*Y*−*δ*_*D*_*D*, the accumulation equation for rural revitalization is *R* = *s*_*R*_*Y*−*δ*_*R*_ *R*, and the accumulation equation for labor is *L* = *nL*. Assuming that economic output can only be distributed between consumption *C* and investment *I*, then *Y* = *C*+*I*, which can be further deduced as *C* = (1−*s*_*D*_−*s*_*R*_)*Y*.

Suppose that the total level of social welfare *U* is determined by social consumption *C*. When the economy reaches equilibrium, $\int_{0}^{\infty }U(C)e^{-\varepsilon t}dt$ is maximized, and *ε* is the discount rate. The constraint is then transformed as:

$$s_{D}Y=\delta_{D}D$$

$$s.t.\{\begin{array}{c} s_{R}Y=\delta_{R}R\\ C=(1-s_{D}-s_{R})Y \end{array}$$ (2)

This creates the following Hamiltonian function:

$$H=U(C)e^{\left(-\varepsilon t\right)}+\lambda (s_{D}Y-\delta_{D}D)+\mu (s_{R}Y-\delta_{R}R)+\pi C$$ (3)

Taking the first-order partial derivatives of the above equations for the digital economy *D* and rural revitalization *R*, respectively, yields:

$$\{\begin{array}{c} \frac{\partial H}{\partial D}=\lambda \cdot s_{D}\cdot \frac{\partial Y}{\partial D}-\lambda \cdot \delta +\mu \cdot s_{R}\cdot \frac{\partial Y}{\partial D}\\ \frac{\partial H}{\partial R}=\lambda \cdot s_{D}\cdot \frac{\partial Y}{\partial R}-\mu \cdot \delta +\mu \cdot s_{R}\cdot \frac{\partial Y}{\partial R} \end{array}$$

Let the first-order partial derivative equal to zero, and further find the conditions for the optimal coupling relationship between the digital economy and rural revitalization at the static optimum:

$$\{\begin{array}{c} \lambda \cdot \delta =\alpha \cdot \frac{Y}{D}(\lambda \cdot s_{D}+\mu \cdot s_{R})\\ \mu \cdot \delta =\beta \cdot \frac{Y}{R}(\lambda \cdot s_{D}+\mu \cdot s_{R}) \end{array}\Rightarrow \frac{D}{R}=\frac{\alpha }{\beta }$$ (4)

The ratio of optimal coupling and coordination between the digital economy and rural revitalization is equal to the ratio of the elasticity coefficients of both. When the digital economy and rural revitalization reach equilibrium in factor flow, the two systems are in a state of coupling and coordination; when either system matches too many factors, the coupling is out of balance. In order to achieve optimal coupling, the factors will be transferred to the other system until the coupling is coordinated. Therefore, there is an optimal coupling and coordination relationship between the digital economy and rural revitalization.

### 2.3 The degree of coupling and the coupling coordination model

The previous section looked at how the digital economy and rural revitalization work together in a complex economic system. This section build a model of how the two work together.


$$\{\begin{array}{c} C_{v}=\sqrt{\frac{{\left(\mu_{1}-\mu_{2}\right)}^{2}}{2}}/\frac{1}{2}(\mu_{1}+\mu_{2})=\sqrt{2(1-C)}\\ C=\sqrt{4\mu_{1}\mu_{2}}/(\mu_{1}+\mu_{2}) \end{array}$$ (5)

*C*_*v*_ indicates the average deviation of the digital economy and rural revitalization; the smaller the value, the smaller the deviation and the stronger the coupling. When *C*_*v*_ = 0, i.e., the coupling degree *C* = 1, the two systems are less deviated from each other, and the coupling is better. The closer the coupling degree *C* is to 0, the worse the coupling level between the two systems. Although the coupling degree can determine the degree of interaction between the digital economy and rural revitalization, the development of the digital economy and the promotion of rural revitalization cannot be entirely consistent. When the values of *μ*_1_ and *μ*_2_ are relatively low, and the scores are similar in a specific region, the use of a coupling degree may lead to the phenomenon of "pseudo-evaluation" and make the evaluation results inaccurate. Following this formula, this paper builds a model that shows how well different parts of the country work together.


$$D=\sqrt{C\times T}其中T=a\mu_{1}+b\mu_{2}$$ (6)

*D* is the coupling coordination degree, *C* is the coupling degree of the digital economy and rural revitalization, *T* is the comprehensive reconciliation index of the digital economy and rural revitalization, which can reflect the level of coordination between the digital economy and rural revitalization. *a* and *b* are coefficients to be determined, *a*+*b* = 1, *a* and *b* are both assigned a value of 0.5. The coupling coordination of the digital economy and rural revitalization is divided into five stages: moderate dissonance, on the verge of dissonance, barely coordinated, moderately coordinated, and highly coordinated. The coordination between the digital economy and rural revitalization was divided into five stages: moderate dissonance, near dissonance, barely coordinated, intermediate coordination, and extreme coordination. When 0<*D*≤0.4, it indicates that the digital economy and rural revitalization are at the stage of moderate dissonance, i.e., the digital economy fails to promote the development of rural revitalization effectively, and rural revitalization fails to transform into the driving force of the digital economy, and the two are at the stage of mutual constraint. When 0.4<*D*≤0.5, it indicates that the digital economy and rural revitalization are at the stage of mutual consent, which means that the digital economy and rural revitalization are at the stage of imminent dysfunction, i.e. the level of coupling and coordination between the two is low, but the power of the digital economy and rural revitalization is decreasing. When 0.5<*D*≤0.6, it means that the digital economy and rural revitalization are at the stage of barely coordinated, and there is more room for improvement in the matching of the two systems in terms of structure and scale. When 0.6<*D*≤0.8, it indicates that the digital economy and rural revitalization are at an intermediate level of coordination, and the digital economy and rural revitalization are at a high level, with a good relationship between the two systems, which is mutually influencing and promoting each other. When 0.8<*d*≤1, it indicates that the digital economy and rural revitalization are at the best and most ideal stage of coupling and coordination. At this point, as the digital economy grows, the level of rural revitalization grows, which in turn helps the digital economy grow even more, and the spiral between the two is getting better.

## 3. Research methodology and construction of the indicator system

### 3.1. Details of the composition of the rural revitalization indicator system

This paper constructs the indicator system for China’s rural revitalization level according to the three-step selection process of indicators. Firstly, based on the reports of the 18th and 19th National Congresses of the Party, the Strategic Plan for Rural Revitalization (2018~2022), the Opinions of the State Council of the Central Committee of the Communist Party of China on the Implementation of the Rural Revitalization Strategy, and the Law on the Promotion of Rural Revitalization, five first-level indicators were selected, namely, prosperous industry, pleasant ecological living, civilized countryside, effective governance, and affluent living. Secondly, 25 secondary indicators were selected, considering the actual situation of measuring rural revitalization. Based on the specific connotation and data availability of the secondary indicators and the comparability of the indicators, the specific measurement formulae for the 25 secondary indicators were formulated. Specifically, rural revitalization in China is the target level, with five primary indicators (subsystems) of prosperous industry, ecological livability, civilized countryside, effective governance, and prosperous living, and five secondary indicators under each primary indicator, covering a total of 25 secondary indicators. The entropy weighting method is used to figure out the weight of each secondary indicator based on the sample data. The weight of the primary indicators is then based on the secondary indicators.

### 3.2 Construction of the evaluation index system for China’s digital economic development

The core concept of this paper in constructing the digital economy development evaluation index system is to, based on the environment of digital governance, with digital economy infrastructure investment, vigorously promote digital industrialization and industrial digital integration development. At present, the following types of indicators are used to measure the level of China’s digital economy’s development: First, the Digital City Development Index is provided by Tencent and the Global Digital Economy Index by Ali Research Institute; second, the indicator system is reconstructed based on the research framework of CITIC Tong Research Institute; and third, the digital economy efficiency coefficient is used as a measurement variable. The indicators of different dimensions of digital economic development all contain useful information on digital economic development, and considering only one or a certain dimension of indicators will lead to a one-sided understanding of digital economic development. Therefore, this paper takes into account the new trends and characteristics of digital economic development and builds a regional digital economic development indicator system in China based on the availability, continuity, reliability, and comparability of existing indicators. As shown in Table 1, the indicator system has four main indicators and 21 other indicators.

**Table 1:** Index system of China’s rural revitalization and digital economy development.

Subsystems	Primary Indicators	Secondary indicators	Indicator measurement formula	Weights
China’s rural revitalization	Industrial prosperity (0.209)	Labour productivity	Value added of primary industries/rural population	0. 029
		Land productivity	Value added of primary sector/total crop area sown	0. 061
		Total machinery power per capita	Total power of agricultural machinery/employees in primary sector	0. 054
		Amount of fertilizer applied per unit of arable land	Agricultural fertilizer use/total crop area sown	0. 027
		Share of primary sector value added	Primary sector increase/GDP	0. 036
	Ecological and livable (0.257)	Sanitary toilet penetration rate	Number of farm households using sanitary toilets/total number of farm households	0. 019
		Piped water penetration rate	Number of farm households using piped water/total number of farm households	0. 021
		Thousands of village health office staff	Number of health technicians/village population	0.102
		Greenery coverage	Area covered by greenery/total village area	0. 068
		Forest cover	Forest area / Total land area	0. 048
	Civilization of the countryside (0.177)	Public library holdings per capita	Public book collection/village population	0. 085
		Cultural station coverage	Number of cultural stations in townships/number of townships	0. 026
		Proportion of expenditure on culture and education	Volume of cultural and educational expenditure/total consumption	0. 015
		Average years of schooling	(Number of illiterates X 1 + number of primary school students X 6 + number of junior secondary school students X 9 + number of high school and secondary school students X 12 + number of college and university students and above X	0. 011
			16) / Total population aged 6 years and over	
		Integrated TV coverage	TV ownership/total number of farm households	0. 040
	Effective governance (0.196)	General public budget	Wages and benefits, goods and services, capital expenditures and other expenditures	0. 042
		Proportion of village improvement carried out	Number of villages under improvement/number of administrative villages	0. 035
		Proportion with village construction planning	Number of villages with construction plans/number of administrative villages	0. 018
		Proportion with township master plan	Number of townships with master plans/total number of townships	0. 012
		Coverage rate of village committees	Number of village committees/number of natural villages	0. 089
	Living well (0.161)	Aged care institutions	Number of elderly service institutions/rural population	0. 044
		Living space per capita	Residential building area/rural population	0. 043
		Income comparison between urban and rural residents	Income of urban residents/income of rural residents	0. 014
		Engel Coefficient	Total food expenditure/total consumption expenditure	0. 016
		Disposable income per capita	Average of rural personal disposable income	0. 043
Digital Economy	Digital Economy Infrastructure (15.09%)	Length of fiber optic cable	Length of long-distance fiber optic cable lines (million kilometers)	0.0186
		Number of cell phone base stations	Number of cell phone base stations in the region (10,000)	0.0256
		Cell phone penetration rate	Total number of telephone sets (including cell phones) / total population of the administrative region × 100 (departments)	0.0073
		Number of Internet broadband access ports	Internet broadband access ports (10,000)	0.0179
		Number of Internet users as a proportion of resident population	Number of Internet users/resident population	0.041
		Number of Internet domain names	Number of Internet domain names (10,000)	0.0404
	Industry Digitization (36.97%)	Digital Inclusive Finance Index	Peking University Digital Inclusive Finance Index	0.128
		Enterprise informatization level	The proportion of enterprises adopting information management (%)	0.129
		Number of courier services	Express delivery volume (million pieces)	0.0073
		E-commerce Sales	E-commerce sales (billion yuan)	0.1053
	Digital Governance (27.86%)	Years of education per capita	Average years of schooling = (number of illiterate people × 1 + number of people with elementary school education × 6 + number of people with junior high school education × 9 + number of people with high school and secondary school education × 12 + number of people with college or university education × 16) / total number of people over 6 years old (years)	0.0363
		Investment intensity of R&D expenditure	The ratio of internal expenditure on R & D to GDP (%)	0.0341
		Number of Digital Economy Enterprises	Information transmission, computer services and software industry, the number of legal persons (units)	0.0393
		Total Technology Contract Transaction	Total turnover of technology contracts (million yuan)	0.0555
		Number of patent applications	Number of invention, utility model and appearance 3 kinds of patent applications (pieces)	0.0371
		Number of granted patent applications	Number of inventions, utility models and appearance 3 kinds of patents granted (pieces)	0.0403
		Digital government level	Number of government websites (pcs)	0.0360
	Digital Industrialization (20.08%)	Digital Industry Employees	Information transmission, software and information technology service industry year-end average number of employees (people)	0.0482
		Total industrial output value of the digital industry	Total industrial output value of communications equipment, computer and other electronic equipment manufacturing industry (billion yuan)	0.0595
		Telecommunications business volume	Total telecommunications business (billion yuan)	0.0365
		Software Industry Revenue	Software business revenue (million yuan)	0.0566

1. Indicators of digital economic infrastructure. A digital economy is an economic form in which new digital technologies are widely used, and the prerequisite for the application of digital technologies is a sound digital economy infrastructure. For example, there are indicators that show how long optical fiber cables are and how many mobile phone base stations there are. There are also indicators that show how many Internet broadband access ports there are and how many Internet users there are.
2. Industrial digitalization indicators. The digitalization of industries brings about increased output and efficiency through the convergence and penetration of ICT products and services in other areas, especially in the three industries. This paper uses the digital financial inclusion index, the level of enterprise informationization, the number of express businesses, e-commerce sales, etc. to measure this.
3. Digital governance indicators. Digital governance is an important guarantee for the healthy and orderly development of the digital economy, covering government, policy, industry, innovation, property rights, corporate governance, and other levels. This paper selects indicators such as the number of years of education per capita, the intensity of investment in R & D expenditure, the number of enterprises in the digital economy, the total amount of technology contract transactions, the number of patent applications, the number of patent applications granted, and the number of government websites (the level of digital government) to measure the level of digital governance.
4. Digital industrialization indicators. Digital industrialization refers to the added value of an information industry characterized by digital technology, including digital technology innovation and digital industrial production, mainly including electronic information manufacturing, information and communication industries, software service industries, and Internet-related industries. This paper uses digital industry employees, the total industrial output value of the digital industry, telecommunications business volume, and software industry revenue to measure digital industrialization indicators.

### 3.3 Data sources and description

This paper uses panel data from 30 provinces in China from 2011 to 2020 as the sample for examination, and in view of the availability and comparability of data, the Tibetan region, as well as Hong Kong, Macao, and Taiwan regions, are not considered for the time being. The data comes mostly from the China Statistical Yearbook, China Rural Statistical Yearbook, China Urban and Rural Construction Statistical Yearbooks, China Population and Employment Statistical Yearbook, and China Population and Employment Statistical Yearbook, among other sources. The data were downloaded from the EPS data platform. Some missing values were filled in by interpolation, and some of the most recent year’s data was manually processed. In order to facilitate the subsequent spatial and temporal variation analysis, the paper divides the 31 provinces of China into four economic regions, namely the eastern region, the central region, the northeastern region, and the western region, according to the 2011 classification method of the National Bureau of Statistics.

### 4.1 An examination of the spatial and temporal characteristics of China’s digital economy and rural revitalization

(1) Overall spatial and temporal characteristics of the digital economy and rural revitalization

**Fig 2:**
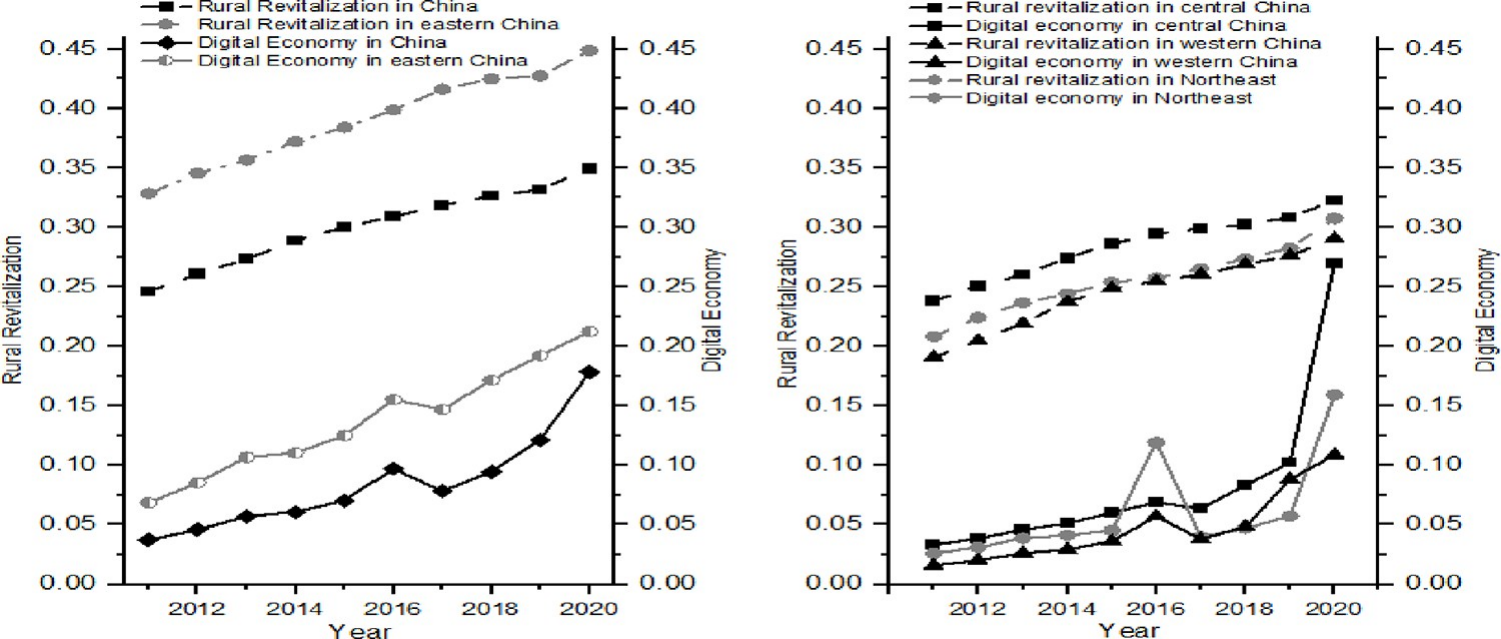
Trends of the digital economy and rural revitalization.

According to the digital economy and rural revitalization index system constructed in Table 1, the digital economy and rural revitalization indices of 30 provinces in China from 2011 to 2020 were first measured by the entropy weight method. Then the coupling coordination degree of China’s rural revitalization and digital economy was calculated according to the coupling degree model and the coupling coordination degree model constructed in the previous section. Fig 2 shows the development trend of the digital economy and rural revitalization across the country and the four major regions, showing the following three characteristics: Firstly, both the digital economy and rural revitalization show a relatively stable upward trend with a noticeable consistent trend, which is the basis for a coupling and coordination relationship between the two. Secondly, the digital economy and rural revitalization have had the same detailed changes. For example, nationwide, there is a similar inflection point in 2019. In the eastern region, the central region, the western region, and the northeastern region, the digital economy and the development of rural revitalization also showed similar fluctuations in 2016 and 2019, indicating an inevitable coupling and coordination relationship between the two on a local scale. Thirdly, the development of rural revitalization is ahead of the digital economy, which reveals that China’s rural revitalization strategy of "prosperous industry, pleasant ecology, civilized countryside, effective governance, and prosperous life" has accelerated the shortcomings in rural development and rapidly improved the quality of rural revitalization development. The digital economy provides more possibilities for developing the rural economy and serving rural revitalization, while rural revitalization provides fertile ground for the digital economy to improve quality and efficiency, innovate and develop. The quality of digital economy development in rural revitalization has been low, which shows that the integration and development of the digital economy is one of the main things that is holding back rural revitalization right now.

(2) Spatial and temporal distribution pattern of the digital economy and rural revitalization

**Fig 3:**
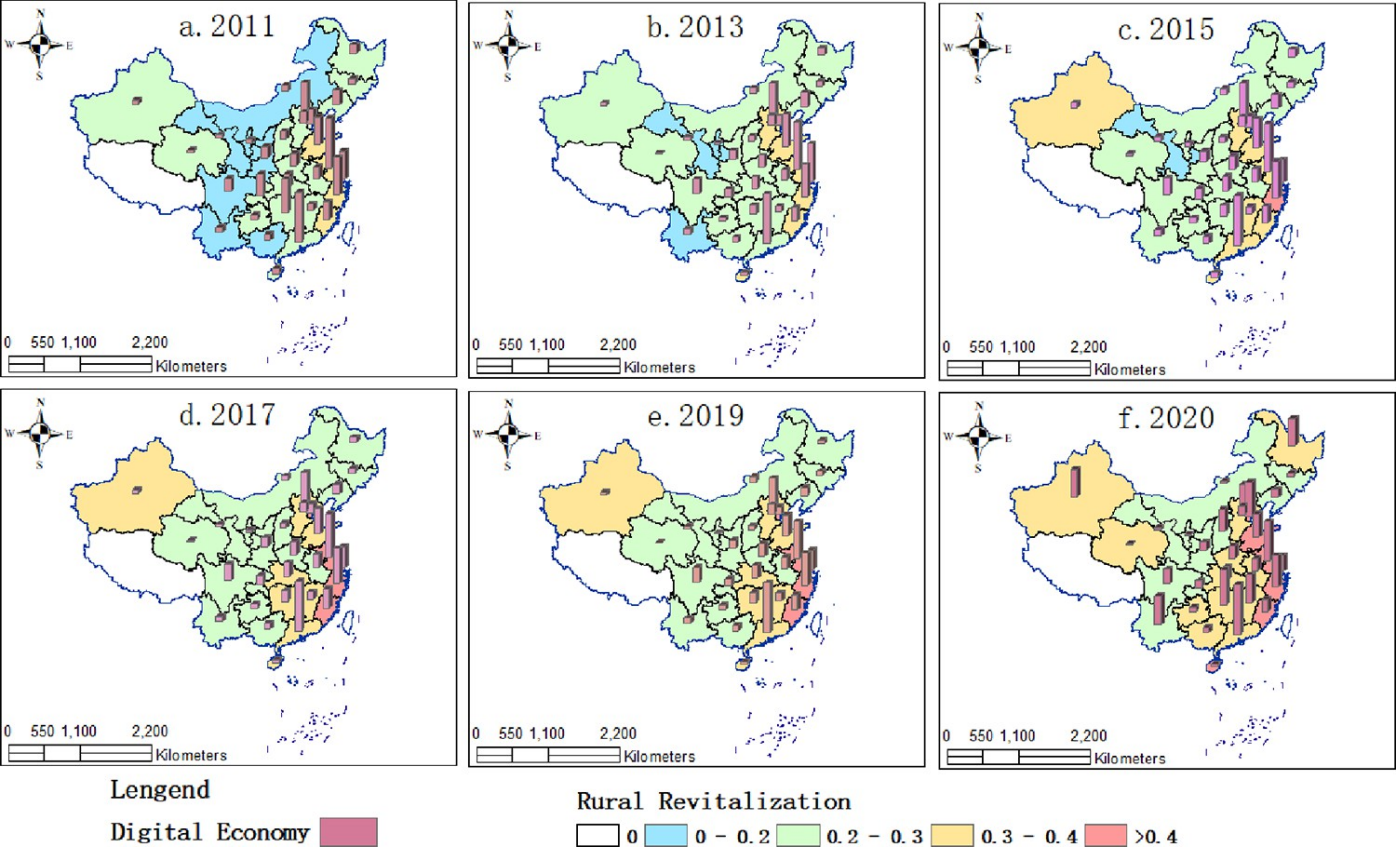
Spatial and temporal distribution of the development level of China’s digital economy and rural revitalization.

The spatial pattern of the digital economy and rural revitalization in China is shown in Fig 3. The main reasons for the rapid development of the digital economy and rural revitalization in all regions of China during the period under review are: firstly, the digital economy development strategy has led to the rapid application and popularization of Internet information technology and e-commerce in China; secondly, the foundation of the digital economy and rural revitalization in most regions of China was relatively weak in 2011, which made it easy to form a high growth trend; and thirdly, with the policy support and practice of the state in fully supporting rural development, all sectors of the country have made every effort to solve the problem of rural revitalization. Furthermore, in practice, especially since the 19th National Congress, which explicitly proposed rural revitalization, various industries have made every effort to address the weak links in rural development, providing a virtual environment for the rapid development of the digital economy and rural revitalization.

As for the digital economy, the average development level of China’s digital economy from 2011 to 2020 was 0.148, growing from 0.088 in 2011 to 0.218 in 2020, achieving leapfrog development. The growth rate is slowing down, indicating that China’s digital economy is becoming more mature as the digital economy strategy steadily advances. There is a clear imbalance in China’s digital economy between regions at the regional level. It is the eastern region that has the most progress in digital economics, followed by the central and northeastern regions. The western region is the least developed in this area.

In terms of rural revitalization, the average level of rural revitalization development in China from 2011 to 2020 was 0.304, which has developed from 0.251 in 2011 to 0.352 in 2020, with an average annual growth rate of 4.53%, indicating that the strategy of poverty eradication and rural revitalization is effective. In terms of spatial distribution, areas with better development prospects for rural revitalization are concentrated on the eastern coast and spreading to the central part of China year by year. Heilongjiang Province and the Xinjiang Uygur Autonomous Region have seen a faster increase in rural revitalization, far higher than their neighboring provinces.

(3) Characteristics of the spatial distribution of the coupling coordination degree between the digital economy and rural revitalization

**Fig 4:**
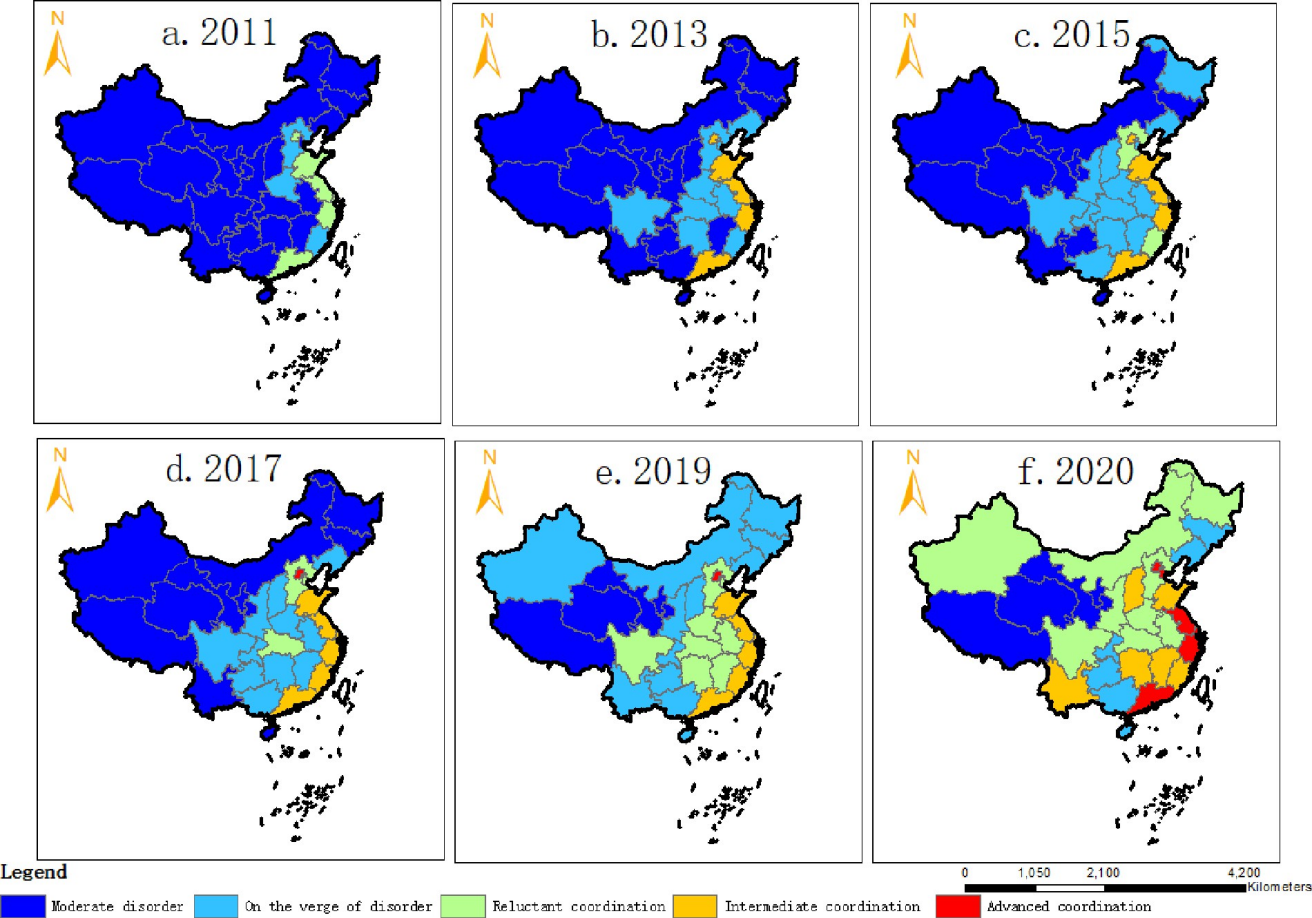
Spatial distribution characteristics of the coupling and coordination degree of digital economy and rural revitalization in China.

The spatial pattern of the coupling coordination degree of China’s digital economy and rural revitalization system is shown in Fig 4. The coupling coordination relationship between China’s digital economy and rural revitalization has gone through three development stages: moderate dissonance, near dissonance, and barely coordinated. The average coupling coordination degree in 2020 was 0.605, which is close to the state of moderate coordination. The analysis of the degree of coupling coordination between the two subsystems of the digital economy and rural revitalization shows that the comprehensive development trend of the two subsystems is consistent. With the rapid improvement of the comprehensive level of the digital economy subsystem, the coupling coordination of the digital economy and rural revitalization subsystem is on an increasing trend, indicating that the digital economy and rural revitalization in China have initially formed the endogenous momentum of synergistic development. However, in terms of development, the overall coupling and coordination between China’s digital economy and rural revitalization are still at a low level. China is at a stage where the rural digital economy lags behind the demand for rural revitalization.

From a regional perspective, the coupling and coordination degree of China’s digital economy and rural revitalization show an apparent characteristic of "strong in the east and weak in the west," and the coupling and coordination degree of the digital economy and rural revitalization from 2011 to 2020 has experienced a gradual diffusion and improvement from the east to the center to the west. In recent years, the coupling and coordination degree between the central and western regions and the northeast region has increased rapidly, but the overall trend of "strong in the east and weak in the west" remains unchanged. The degree of coupling and coordination between China’s digital economy and rural revitalization shows an obvious "polarization and balance" phenomenon but tends to develop in a balanced manner in the same echelon. At present, Beijing, Shanghai, Zhejiang, Tianjin, Jiangsu, and Guangdong, as polarized regions, are the regions with the best development of coupling and coordination between inclusive digital finance and rural revitalization. Especially Beijing, Shanghai, and Zhejiang, whose coupling and coordination level has reached a high coordination stage, forming a gap with other regions in China and showing a particular polarized spatial pattern. In contrast, the difference in coupling and coordination levels among other regions is insignificant. In recent years, all regions have developed from varying degrees of dissonance to intermediate levels of coordination, from moderate dissonance in 2011 to reaching intermediate levels of coordination by 2020, and have made great strides. Overall, there is a balance between the non-polarised regions.

(4) Examine the coupling and coordination constraints between the digital economy and rural revitalization

The "Zhou’s Constraints Identification Index" is proposed and constructed for the first time to identify and visualize the bottlenecks and critical areas that restrict the coupling and coordination between China’s digital economy and rural revitalization. The specific index model is as follows:

$$\{\begin{array}{c} Cons_{it}=U_{1it}/U_{2it}\\ Cons_{i}=(\sum_{t=1}^{T}U_{1it}/T)/(\sum_{t=1}^{T}U_{2it}/T)\\ Cons_{t}=(\sum_{i=1}^{N}U_{1it}/N)/(\sum_{i=1}^{N}U_{2it}/N) \end{array}$$ (7)

Where, *U*_1*it*_ is the level of the digital economy subsystem in the region *i* and year *t*. *U*_2*it*_ and is the level of the rural revitalization subsystem in the region *i* and year *t*. *Cons* denotes the Zhou’s constraint identification index of China’s digital economy and rural revitalization, the value of which is in the range of (0,+∞), *Cons* = 1 indicating that the two systems have a slight deviation and the coupling is good. If it is less than 1, it means that the development level of China’s digital economy is lagging behind the demand for rural revitalization, and the farther the deviation from 1, the more serious the lag is, then the digital economy becomes a constraint to the coupling of the two. If the value *Cons*_*it*_ is greater than 1, it means that the development level of China’s digital economy is over-saturated relative to the demand for rural revitalization, and the farther the deviation from 1, the more serious the over-saturation is, then rural revitalization becomes a constraint to the coupled and coordinated development of the two.

**Fig 5:**
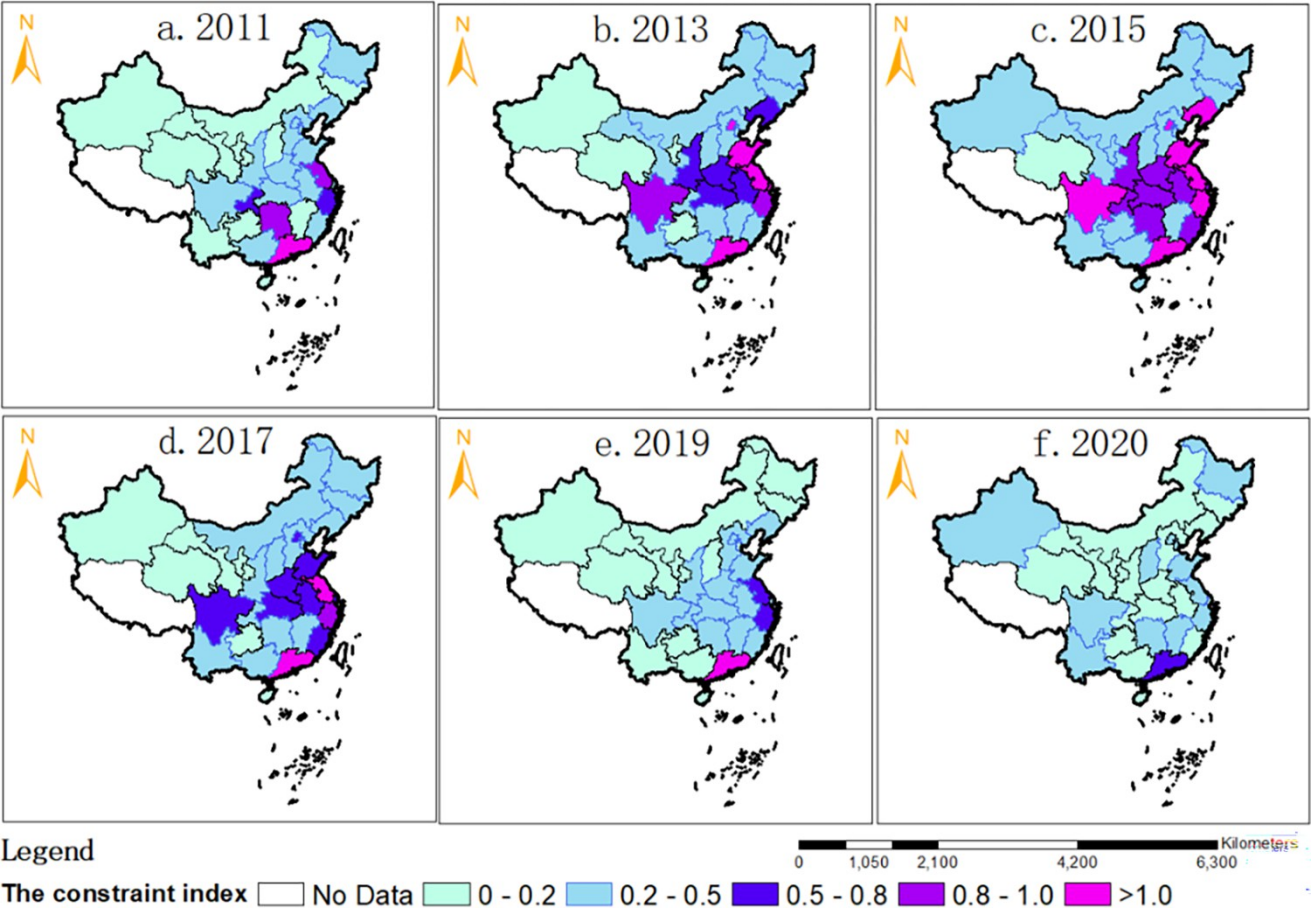
Spatial distribution of Zhou’s constraint identification index in China.

The spatial pattern is shown in Fig 5: From 2011 to 2015, China generally showed that the development level of the digital economy lagged behind the revitalization of the countryside. However, the growth rate of the digital economy was faster than that of the revitalization of the countryside. The digital economy relative to countryside revitalization has become a bottleneck, except for Guangdong and Shanghai, oversaturated, and Jiangsu and Hunan, close to coordination in 2011. Guangdong and Shanghai are oversaturated, while Jiangsu and Hunan are close to coordination. All other regions have different degrees of digital economy lagging, which has become a bottleneck for rural revitalization and urgently needs to develop digital economy assistant rural revitalization vigorously. Digital economy development is faster than rural revitalization, and the constraints of China’s digital economy on rural revitalization have generally eased from 2011 to 2015. The outcome reflects the addition of Beijing, Liaoning, Shandong, Jiangsu, Zhejiang, and Sichuan to the over-saturated areas of the digital economy in Guangdong and Shanghai in 2015 near-coordinated areas of Hunan, Fujian, Hubei, Anhui, Henan, Shaanxi, and Chongqing.

From 2017 to 2020, the development of the digital economy was accompanied by a more rapid enhancement of rural revitalization, especially after the 19th National Congress put forward the rural revitalization strategy, and a series of policies and measures were introduced in various regions, which rapidly promoted the enhancement of rural revitalization. The level of development of the digital economy in that period is increasingly lagging behind the needs of rural revitalization development and has become an essential constraint to rural revitalization. The result reflects the severe degree of digital economy constraints in the development of rural revitalization in all regions of China in 2020. How to break the situation that the development of the digital economy lags behind the needs of rural revitalization development is an urgent task.

### 4.2 Spatial differences and decomposition of the coupling and coordination degree between the digital economy and rural revitalization

According to the Dagum Gini coefficient and decomposition method, the overall differences in the coupling and coordination degree of the digital economy and rural revitalization in China are decomposed into three parts: intra-group differences refer to the differences within the East, Central, West, and Northeast regions; inter-group differences refer to the differences between East-Central, East-West, East-Northeast, Central-West, Central-Northeast and West-Northeast regions. Super-variance density reflects the contribution of cross-over between different regions to the overall differences.

(1)Overall Differences

**Fig 6:**
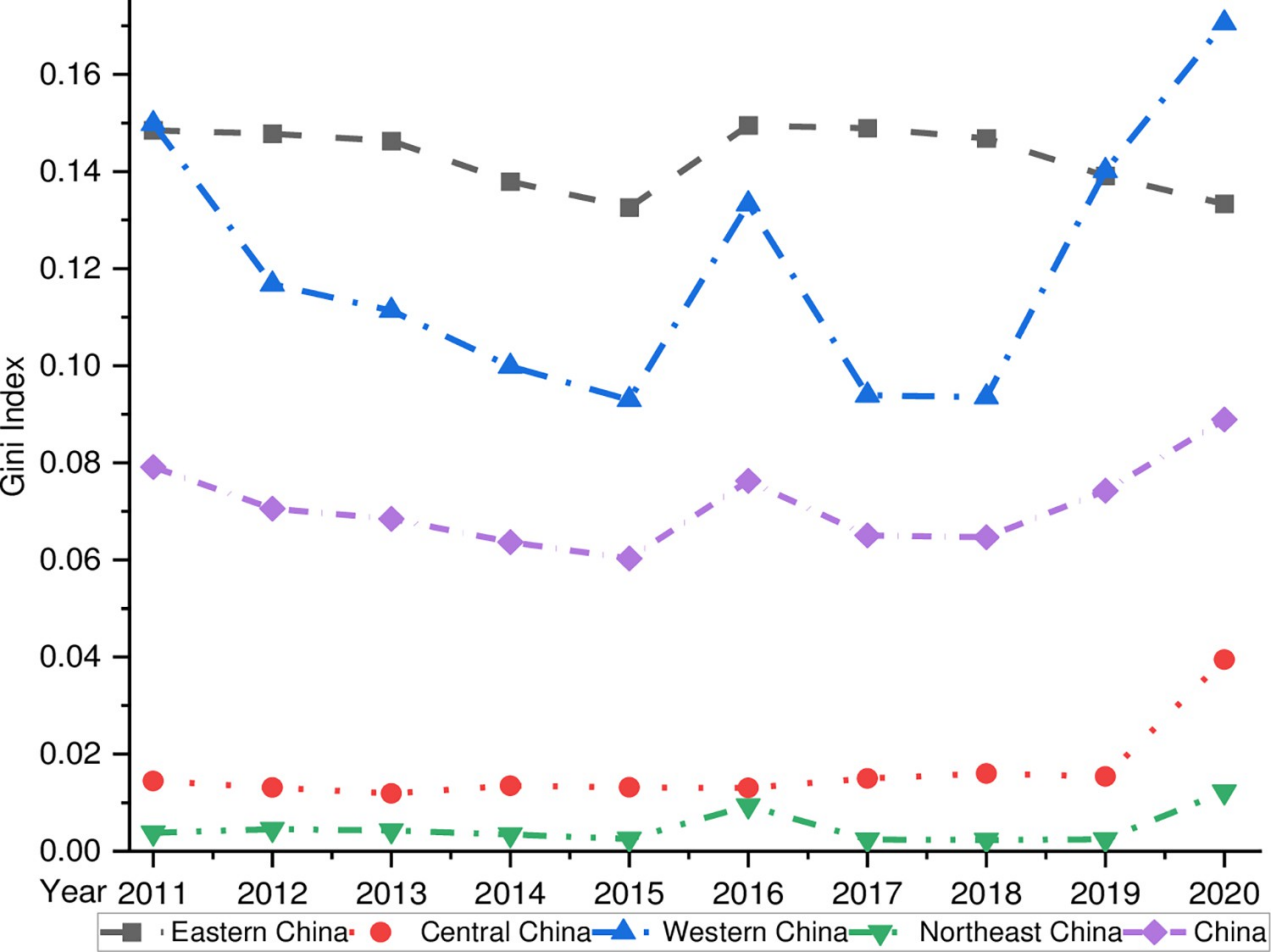
Overall Gini coefficient.

Fig 6 shows the overall Gini coefficient of the coupling coordination between China’s digital economy and rural revitalization and the intra-regional Gini coefficient trend of the four major regions. It can be seen that the overall Gini coefficient of China’s coupling and coordination of digital economy and rural revitalization is on a fluctuating upward trend, from 0.079 in 2011 to 0.088 in 2020, with an average annual growth rate of 1.37%, which indicates that the spatial differences of China’s coupling and coordination of digital economy and rural revitalization are generally on a widening trend. Specifically, from 2011 to 2015, the overall Gini coefficient decreased continuously from 0.079 to 0.063, with an average annual growth rate of -5.646%, indicating the spatial variation in the degree of coordination between China’s digital economy and rural revitalization has been expanding. However, the Gini coefficient rose sharply from 0.060 to 0.076 in 2016, indicating the spatial difference of coupling and coordination between the two was changing in that period.

(2)Intra-regional differences

Fig 6 shows that there is a lot of variation in the level of coordination between the digital economy and rural revitalization in the four major regions of China.

Firstly, the intra-regional Gini coefficient is highest in the eastern region, which shows a trend of "slowly decreasing-rapidly increasing-slowly decreasing." The trend means that the intra-regional differences in how the digital economy and rural revitalization work together in the eastern region are more significant than in the other three regions. The intra-regional differences in the degree of coordination between the digital economy and rural revitalization in the eastern region show a significant spatial convergence. The result is related to the large differences in the level of green innovation among cities in the eastern region. Specifically, Beijing, Shanghai, Zhejiang, and Guangdong in the eastern region have the highest levels of digital economic activity and rural revitalization in China. In contrast, Hebei Province and Hainan Province, both in the eastern region, have a lower digital economy and rural revitalization level, causing a big difference in the eastern region and a more significant Gini coefficient.

Secondly, although the intra-regional Gini coefficient of the western region is lower than that of the eastern region, it has the fastest growth rate, from 0.149 in 2011 to 0.191 in 2020, with an average annual growth rate of 1.538%. The Gini coefficient of the coupling of the digital economy and rural revitalization in the western region catch up with the eastern region at a breakneck speed and finally reach and surpass the Gini coefficient of the eastern region in 2019. Thirdly, the Central and Northeastern regions are the most critical.

Third, the intra-regional Gini coefficients of the central and northeastern regions have long been the lowest among the four regions, indicating that they have minor intra-regional differences but with apparent phase characteristics. After China’s 19th Party Congress, the Gini coefficient rose because of rural revitalization and the digital economy policies, which has led to a widening gap between the two regions.

(3)Regional Differences

**Fig 7:**
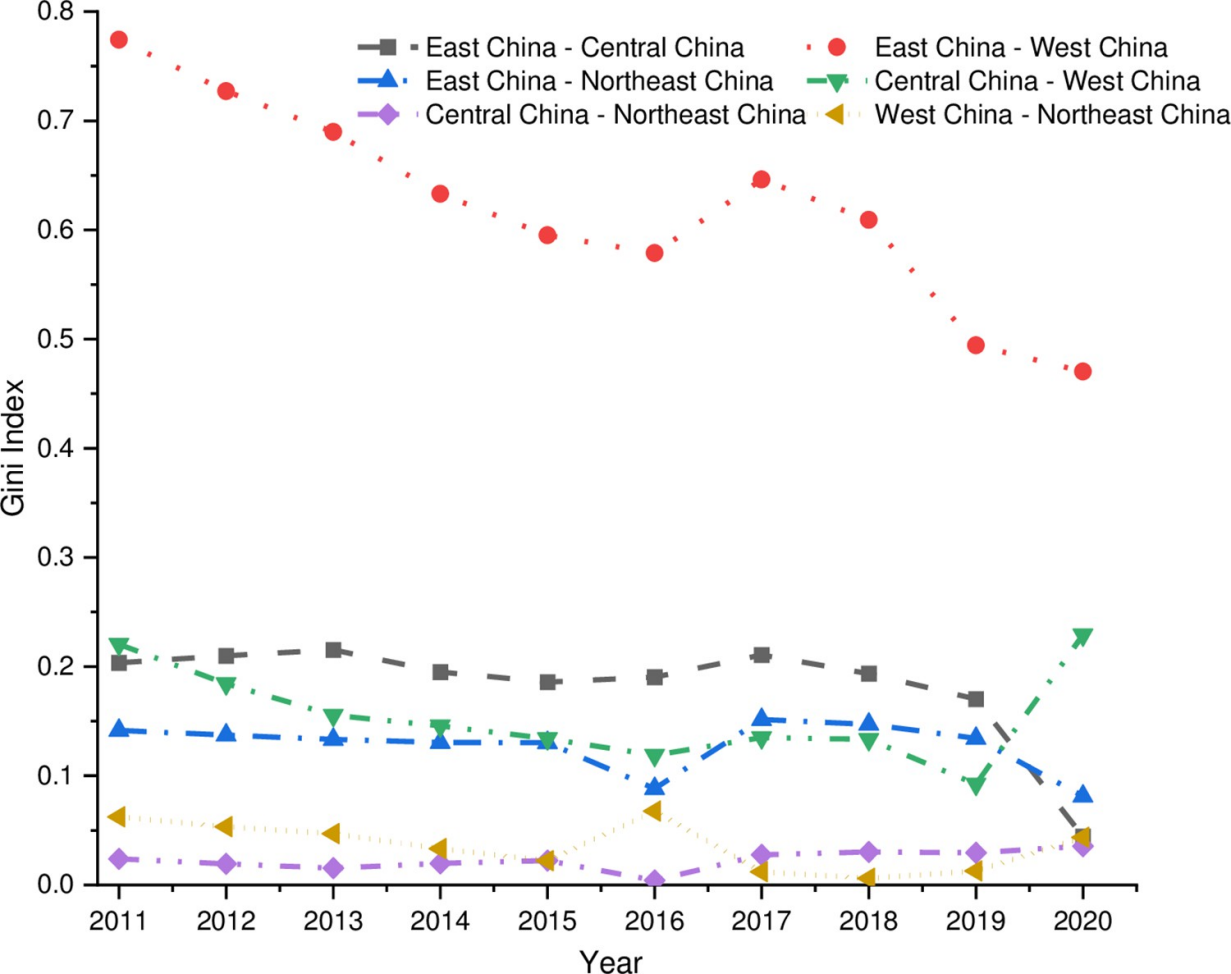
Inter-regional Gini coefficient.

As shown in Fig 7, the inter-regional differences between the digital economy and rural revitalization indicate the magnitude and trend of the differences.

Firstly, from the perspective of the magnitude of the difference, the Gini coefficient between the eastern and western regions is the largest, with an intra-group age value of 0.622, followed by the eastern and central regions, with an intra-group mean value of 0.182. The intra-group mean value in the central and western regions is 0.155, and 0.128 in the eastern and northeastern regions. The differences between the eastern and western regions, the eastern and central regions, the central and western regions, and the eastern and northeastern regions are insignificant. The degree of coordination between the digital economy and rural revitalization differs most between the eastern and western regions, the eastern and central regions, the central and western regions, and the eastern and northeastern regions. In addition, the Gini coefficients between the central and northeastern regions and the western and northeastern regions are the smallest, with mean values of 0.108 and 0.110, respectively. The inter-regional differences are driven by the four inter-regional differences between the eastern and western regions; the eastern and central regions, the central and western regions, and the eastern and northeastern regions.

Secondly, analyzing the changing trend of the gap and the average annual decline rate, we can see that the gap between the eastern and central regions, the eastern and western regions, the eastern and northeastern regions, and the western and northeastern regions has been decreasing, with an average annual growth rate of -8.69%, -4.36%, -4.73%, and -3.35%, respectively, showing the inter-regional convergence trend. The gap between the eastern and central regions has the fastest decline rate. The fastest rate is the average annual growth rates of the Gini coefficients between the central-western, and central-northeastern regions are 0.43% and 5.38%, respectively, after 2019, which indicates that the regional differences in the coordination of the digital economy and rural revitalization in China will be driven by the combination of the central-western and central-northeastern regions for a certain period in the future.

(4)Variation sources

**Fig 8:**
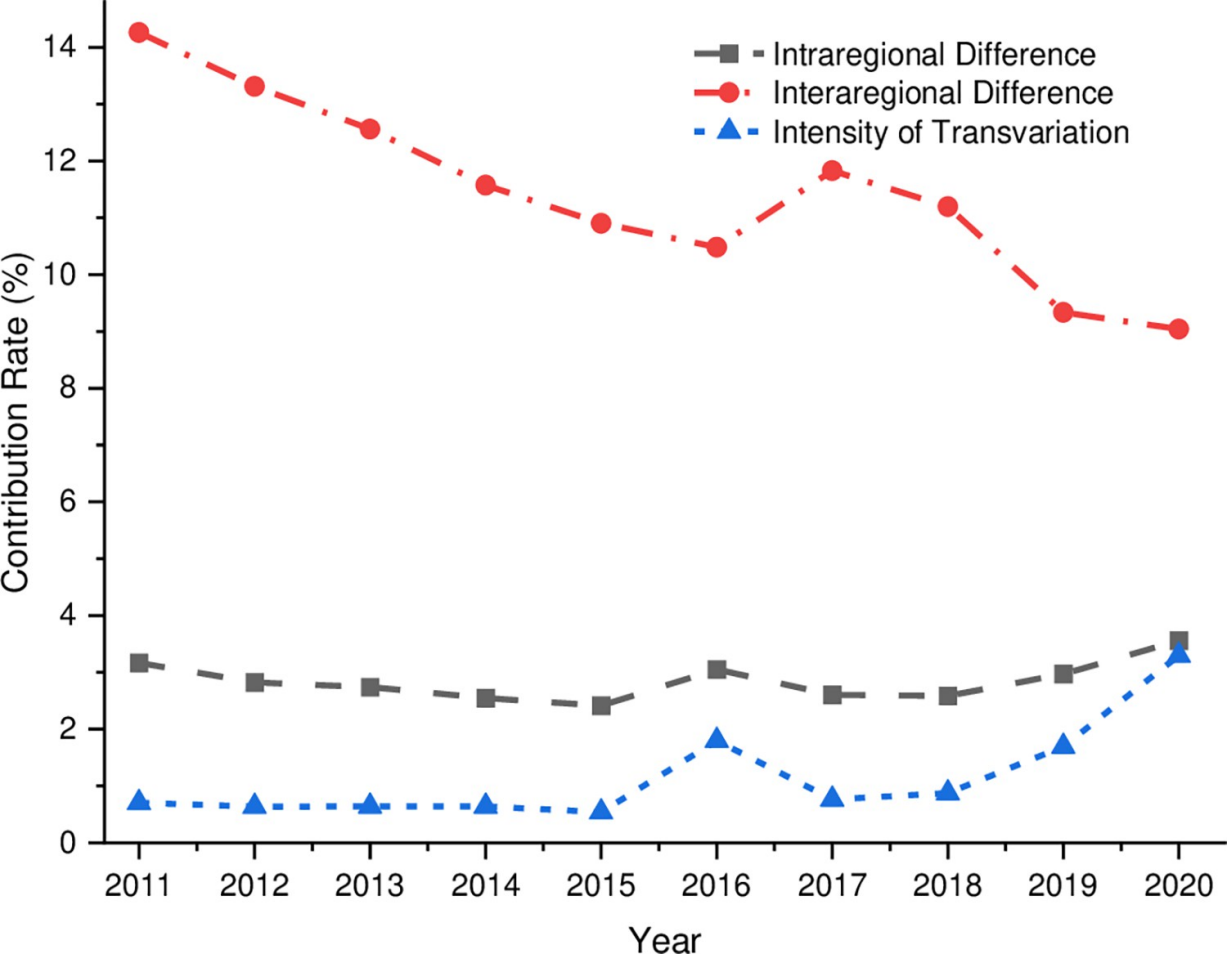
Breakdown of sources of variation.

As shown in Fig 8, the contribution of inter-regional variation, intra-regional variation, and hyper-variance density to the coupling and coordination degree of China’s digital economy and rural revitalization is significant. During the examination period, the contribution of inter-regional differences to the overall variation in the coupling coordination degree has been dominant, followed by intra-regional differences, and the contribution of hypervariable density is the smallest, which indicates that the primary source of the overall variation in the coupling coordination degree of China’s digital economy and rural revitalization is inter-regional differences. The contribution rate trend shows that the inter-regional contribution rate trend is relatively stable and shows a convergence trend. The intra-regional and super-variable density contribution rate trends are slowly increasing, but they are still much smaller than inter-regional differences. The trend suggests that inter-regional variation will still play a big role in the overall change in the next few years.

## 5. Assessment of the degree of coupling and coordination between the digital economy and rural revitalization

### 5.1. Spatial correlation analysis

**Fig 9:**
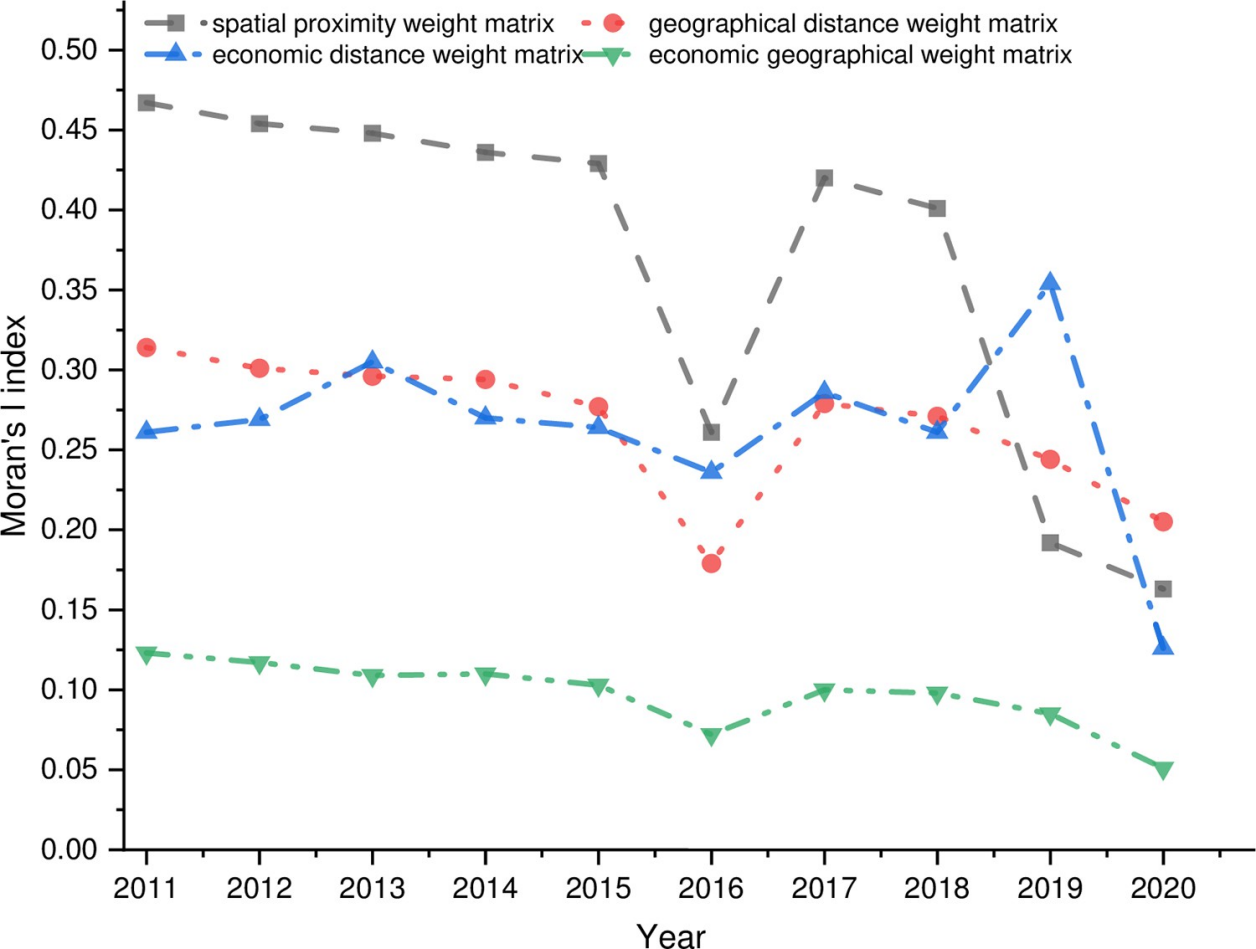
Moran test for different spatial weight matrices.

In order to examine the spatial dependence and spillover of the coupling and coordination degree between the digital economy and rural revitalization in different regions, the spatial proximity weight matrix, geographic distance weight matrix, economic distance weight matrix, and economic geographic weight matrix were constructed in this paper. The spatial Moran’s I index of the coupling and coordination degree of the digital economy and rural revitalization in 1999–2019 under these four spatial weight matrices was calculated, and the results are shown in Fig 9. The results show that the global Moran’s I index is significantly positive during the period under consideration, indicating that the level of coupling and coordination between the digital economy and rural revitalization is influenced by the level of coupling and coordination of neighboring areas and that the spatial distribution pattern of coupling and coordination between the digital economy and rural revitalization is high-high and low-low. From the values of Moran’s I index, except for the spatial proximity weight matrix and the economic distance weight matrix in 2000, which are significant at the 5% level, all the others are positive at the 1% level, indicating that the positive spatial correlation between the digital economy and rural revitalization coupling and coordination is relatively stable. The Moran’s I index of the spatial proximity weight matrix is stable between 0.17 and 0.47, and the Moran’s I index of the geographic distance weight matrix and economic distance weight matrix is stable between 0.15 and 0.32. So, geographic distance and economic distance are essential elements in the coupling and coordination of the digital economy and rural revitalization, and it is necessary to conduct differentiated research at economic and geographic scales. In this paper, the economic-geographic spatial weight matrix is selected for further study because it integrates the stable influence of economic and geographical factors on the spatial pattern, and the Moran’s I index is stable between 0.05 and 0.15, which is lower than the other three spatial weight matrices. The economic-geographical spatial weight matrix makes the empirical results robust.

### 5.2 Absolute β-convergence

The models were screened by the LM test, Hausman test, and fixed effects test to analyze the absolute β-convergence of the coupled development of the digital economy and rural revitalization in the whole country and the eastern, central, western, and northeastern regions. As shown in Table 2, the Robust LM-error test and the LR-SDM-SEM test are not significant under the economic geographical weight matrix, indicating that the spatial Durbin model can be degraded to a SAR model. The Hausman test rejects the original hypothesis at the 1% significance level, indicating that a fixed-effects model should be used. The individual and time effects were tested by LR-both-ind and LR-both-time, respectively, both of which rejected the original hypothesis at the 1% significant level, so the model uses a time-area double fixed effect. The general model was checked out by diagnostic tests. In this paper, the SAR model with two-way fixed effects was used to test the beta convergence process of the coupling degree of China’s digital economy and rural revitalization, as well as the beta convergence process of the beta convergence process.

**Table 2:** Model selection test structure.

Diagnostic Tests	Value	P-Value	Value	Value	P-Value
LM-lag	4.424	0.035	LR-both-time	78.96	0.000
Robust LM-lag	2.907	0.088	LR-SDM-SAR	3.07	0.079
LM-error	3.056	0.080	LR-SDM-SEM	0.02	0.878
Robust LM-error	1.538	0.215	Wald-SAR	26.16	0.000
Hausman	34.23	0.000	Wald-SEM	16.11	0.000
LR-both-ind	44.30	0.000			

Table 3 shows that the absolute β convergence coefficients of the national, eastern, central, western, and northeastern regions are all negative, except for the central region, which is significantly non-zero at the 1% level, indicating that there is significant spatial convergence in the degree of coupling and coordination between the digital economy and rural revitalization in the national and the four regions. The absolute value of the β convergence coefficient is West > National > East > Northeast > Central, implying that the western region converges faster than the east-central and northeast regions because of the digital economy’s development level and rural revitalization. The western region generally has a low convergence rate. With the continuous promotion of the construction of ecological civilization in the Yellow River Basin and the introduction of policies to support rural areas in the West, the development level of the digital economy and rural revitalization in the western region has rapidly improved, and the spatial convergence has shown a higher marginal effect. At the same time, there are still huge differences between the digital economy and rural revitalization in the east and the west. Under the siphoning effect, resources in the central and western regions continue to flow to the east, which may lead to a rapid convergence of the spatial coordination between the digital economy and rural revitalization in the west to a low level of equilibrium.

**Table 3:** Regression result.

	Whole Country	Eastern Region	Middle Region	Western Region	Northeast Region
CCD	-0.800***	-0.560***	-0.143	-0.981***	-0.368**
	(-0.08)	(-0.16)	(-0.21)	(-0.13)	(-0.16)
rho	-1.123***	-0.424*	-1.872***	-0.637**	-0.959***
	(-0.26)	(-0.22)	(-0.24)	(-0.32)	(-0.13)
sigma2_e	0.007***	0.003***	0.002***	0.007***	0.003***
	(0.00)	(0.00)	(0.00)	(0.00)	(0.00)
Province FE	YES	YES	YES	YES	YES
Year FE	YES	YES	YES	YES	YES
*R-squared*	0.1942	0.1196	0.5609	0.2022	0.2009
Log-likelihood	287.9148	135.9762	78.2932	103.7193	29.5924

Note: The numbers in parentheses below the regression coefficients are robust standard errors, and *, **, and *** indicate significance at the 10%, 5%, and 1% levels,respectively.

### 5.3 Relative convergence

Conditional beta convergence refers to the inclusion of a set of control variables that have an impact on the degree of coordination of the coupling of the digital economy and rural revitalisation and are generally more realistic. Drawing on Elhorst (2014) [40], the Hausman test confirms the applicability of a two-way fixed effects model for time and area, and both the spatial LR test and the Wald test confirm the spatial autoregressive model (SAR). In view of this, the spatial conditional β convergence was tested according to the spatial autoregressive model of Lesage (2008) [41], as in Eq 8:

$$ln\;\left(\frac{D_{i,t+1}}{D_{i,t}}\right)=\alpha +\beta ln\;\left(D_{i,t}\right)+\rho \sum_{j=1}^{N}\;w_{ij}ln\;\left(\frac{D_{j,t+1}}{D_{j,t}}\right)+\gamma ln\;X_{i,t+1}+u_{j}+v_{t}+\varepsilon_{it}$$ (8)

Where λ is the spatially lagged regression coefficient, *W*_*ij*_ is the spatial weight matrix, *X*_*i*,*t*_ is the set of control variables, *u*_*i*_ and *v*_t_ are personal and temporal fixed effects, respectively, which can effectively control for unobservable regional and temporal trend factors that do not vary over time, and *ε*_*it*_ is the error term. After adding the control variables, if *β* is significantly less than 0, then there is conditional spatial convergence, and vice versa, there is spatial divergence.

Combining the development of the digital economy and the actual situation of rural revitalization in China, the level of financial support to agriculture, industrial structure, level of inclusive financial development, urban-rural income gap, and economic development level was identified as the influencing factors. Details are shown in Table 4

**Table 4:** Variables and descriptions.

Variables	Variable symbols	Variable descriptions
Degree of coupling coordination	*D*	Degree of coupling and coordination
Financial support to agriculture	*gov*	financial support for agriculture, forestry and water affairs in total fiscal expenditure
Financial Inclusion Development	*fin*	Peking University Digital Inclusive Finance Index
Industrial structure	*indu*	Share of primary industry output in GDP
Level of urbanisation	*urban*	Non-agricultural population as a proportion of total population
Economic Development	*gdp*	Real GDP per capita

Based on the β absolute convergence analysis model, five control variables, including the level of financial support to agriculture, industrial structure, the level of inclusive financial development, the urban-rural income gap, and the level of economic development, were added to obtain the conditional β convergence estimation results of the coupling coordination degree between the digital economy and rural revitalization for the whole country and the four regions. As shown in Table 5, the conditional β convergence coefficients of the national, eastern, and western regions are all significantly negative, which proves that there is a significant conditional convergence process in the above regions, and the degree of coordination between the digital economy and rural revitalization will converge to their respective steady-state levels under the influence of different influencing factors. Spatial differences tend to decrease over time, and there is a "catch-up effect" for the lagging regions. In terms of the convergence trend, the western region and the whole country have the fastest convergence rate, followed by the eastern region, while the central and northeastern regions have weak convergence. Therefore, there is both absolute and conditional convergence across the country as a whole and the four regions, which also indicates that, in the long run, the spatial differences in the degree of coupling and coordination between the digital economy and rural revitalization in China are gradually decreasing, and this trend is relatively stable.

**Table 5:** Model estimation results.

	The Whole Country		Eastern Region	Middle Region	Western Region	Northeast Region
D	-0.607*** (-0.12)	-0.304* (-0.16)		-0.200 (-0.18)	-0.619*** (-0.20)	-0.636 (-0.67)
gov	-0.093 (-0.07)	-0.461*** (-0.10)		-0.504*** (-0.15)	-0.221 (-0.18)	-0.514** (-0.26)
indu	-0.019*** (-0.01)	-0.009 (-0.01)		-0.012 (-0.01)	-0.040*** (-0.01)	-0.048 (-0.03)
fin	-0.030** (-0.01)	-0.013 (-0.01)		-0.005 (-0.05)	-0.045** (-0.02)	-0.049 (-0.09)
gdp	-0.019*** (-0.01)	-0.009 (-0.01)		-0.012 (-0.01)	-0.040*** (-0.01)	-0.048 (-0.03)
urban	0.777* (0.42)	1.000*** (0.37)		0.793 (1.40)	1.150 (1.92)	4.753 (3.71)
rho	-1.122*** (-0.26)	-0.347* (-0.21)		-1.953*** (-0.21)	-0.724** (-0.32)	-1.014*** (-0.12)
sigma2_e	0.006*** (0.00)	0.003*** (0.00)		0.001*** (0.00)	0.006*** (0.00)	0.003*** (0.00)
Province FE	YES	YES		YES	YES	YES
Year FE	YES	YES		YES	YES	YES
*R* ^2^	0.1926	0.1181		0.1071	0.3820	0.1781
LogL	295.7233	151.020		89.4761	112.3908	31.8718

Note: The numbers in parentheses below the regression coefficients are robust standard errors, and *, **, and *** indicate significance at the 10%, 5%, and 1% levels,respectively.

The effects of control variables on the convergence of the coupling coordination, specifically, First, the level of financial support for agriculture generally affects the convergence of the coupling coordination degree between China’s digital economy and rural revitalization, especially significantly contributing to the convergence of the coupling coordination degree in the eastern, central, and northeastern regions. Financial support to agriculture is the basis for improving the coupling and coordination between China’s digital economy and rural revitalization. That each region should get more money from the government to keep looking for new ways to grow the rural digital economy and make sure that there are enough materials and technology to help revitalize the countryside.

Secondly, the impact of urbanization on the degree of coupling and coordination between the digital economy and rural revitalization varies from region to region, with urbanization hindering the convergence of the degree of coupling and coordination in the national and eastern regions and positively affecting the central, western, and northeastern regions. This phenomenon may be because the current urbanization process in China is a "Great Leap Forward" type of low-quality development, which is obsessively pursuing the growth of the urban population share. At the same time, farmers are encouraged to move to the cities without being properly resettled, which slows down the revitalization of the country.

Thirdly, the level of financial inclusion development significantly contributes to the convergence of the digital economy and rural revitalization coupling in the national and western regions, while the impact on the central and northeastern regions is positive but not significant. In order to improve the quality of rural revitalization, there must be a smooth flow of money and a rational allocation of funds. This should be done to solve the problem of wasted money in the eastern region and to ease the contradiction of total money in the less developed western region.

Fourth, GDP per capita significantly contributes to the convergence of the coupling coordination between the digital economy and rural revitalization to high values in both national and western regions. With the increase in per capita income, residents will have more opportunities to borrow the Internet, stimulating platform consumption and driving the digital economy’s development. At the same time, rural social welfare and the rural ecological environment will also improve, which will promote rural revitalization.

Fifthly, the coefficient of foreign investment is significant at the 5% level. The introduction of foreign investment currently brings advanced digital technology and digital management experience, generating a positive spillover effect and promoting the improvement of the coupling and coordination between domestic digital economic development and rural revitalization.

### 5.4 Sensitivity testing

(1) The effects of various weight settings

**Fig 10:**
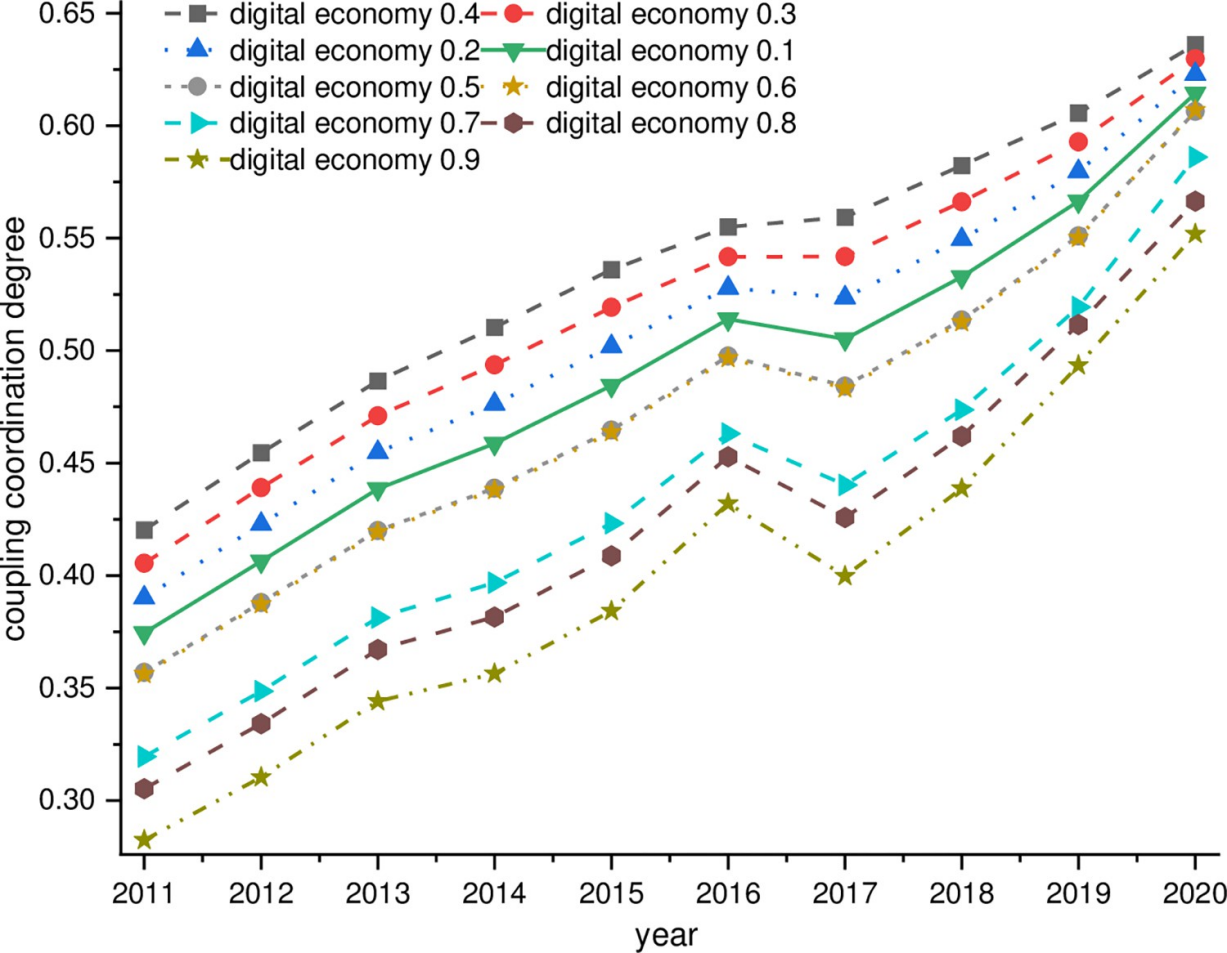
Effect of various weight settings.

By setting the weights of the digital economy and rural revitalization to a total of nine weight combinations from 0.1 to 0.9, respectively, the calculated dynamics of the coupling and coordination degree of the digital economy and rural revitalization are shown in Fig 10. The influence of different weights on the degree of coordination between the digital economy and rural revitalization in China is undeniable, with the weighting of rural revitalization-digital economy 0.9–0.1 gradually changing to rural revitalization-digital economy 0.1–0.9, the level of coordination between the digital economy and rural revitalization gradually increasing and then gradually decreasing. When the weight of the digital economy reaches 0.4, the level of coupling and coordination reaches its maximum. The reasonable weight of the digital economy in the process of coupling and coordinating the development of China’s digital economy and rural revitalization is between 0.4 and 0.5, which also proves that the weight of 0.5 for the digital economy and 0.5 for rural revitalization set in this paper is reasonable.

(2) Robustness of β-convergence

Therefore, the combination of 0.4 digital economy and 0.6 rural revitalization weights was selected to calculate the degree of coordination between China’s digital economy and rural revitalization, and the β convergence model was regressed. The results show that when the weight of the digital economy is set at 0.4, there is still a significant absolute β convergence and conditional β convergence process, and the influence of the factors in the conditional β convergence does not change significantly, which also indicates that the study results are robust.

## 6. Conclusions and recommendations

Based on China’s provincial data from 2011 to 2020, an evaluation index system for the coupled and coordinated development of China’s digital economy and rural revitalization, including 46 indicators of the digital economy and rural revitalization subsystem, was constructed, and the entropy weight method, coupling coordination model, constraint identification index, Dagum Gini coefficient decomposition method, and panel spatial econometric model were combined to analyze the measures, constraints, dynamic evolution of distribution, regional differences, sources, and convergence of the coupled and coordinated development of China’s digital economy and rural revitalization.

First, both the digital economy and rural revitalization grew at a steady rate during the study period. The quality of rural revitalization, in general, was much better than that of the digital economy, which lacked the demand for the digital economy in rural revitalization in most periods and regions and became a bottleneck for rural revitalization. The constraints of the digital economy have been gradually eased. In 2015, cities like Guangdong, Shanghai, Beijing, Liaoning, and Shandong, as well as provinces like Shandong and Jiangsu, saw an overabundance of the digital economy over rural revitalization. However, with the introduction of the rural revitalization strategy in the 19th Party Congress, governments at all levels have introduced responsive policies to promote rural revitalization. The lagging constraints of the digital economy on the development of rural revitalization from 2017 to 2020 have gradually come to the fore again, with all regions experiencing varying degrees of digital economy constraints in 2020. According to the degree of digital economy constraints on rural revitalization development in different regions, it is imperative to adjust the government’s investment in the digital economy in each region.

Second, in terms of spatial differences and decomposition, the spatial differences in the degree of coupling and coordination between the digital economy and rural revitalization in China are gradually decreasing in the eastern region, increasing in the western region year by year, and relatively stable in other regions, gradually showing a spatial convergence and balanced development trend. The contribution of inter-regional differences to the overall variation in the coupling and coordination degree has been dominant, followed by intra-regional differences, with the most negligible contribution from hyper-variance density, which indicates that the primary source of the overall variation in the coupling and coordination degree of China’s digital economy and rural revitalization is inter-regional differences. The contribution rate trend shows that the inter-regional contribution rate trend is relatively stable and shows a convergence trend. The intra-regional and super-variable density contribution rate trends are slowly increasing, but they are still much smaller than inter-regional differences. The results show that inter-regional variation will still play a big role in the overall change in the next few years.

Thirdly, through the spatial β convergence test and the analysis of influencing factors, the spatial weight matrix shows a significant spatial conditional β convergence characteristic of the coupling coordination degree of China’s digital economy and rural revitalization. Under different influencing factors, the degree of coordination between the digital economy and rural revitalization converges to their respective steady-state levels in each region. The spatial difference tends to decrease over time, and there is a "catch-up effect" for the lagging regions. In terms of convergence, the western region and the whole country have the fastest convergence rate, followed by the eastern region, while the central and northeastern regions have a weaker convergence.

These spatial and temporal differences and bottlenecks in the digital economy are caused by geographic location, environmental differences, and government priorities, which can be adapted to the actual situation in each region. The developed provinces and cities on the eastern coast of China have a better digital economic environment, which is inherently superior for implementing the "digital countryside" strategy. Most of the central provinces and cities are paying more attention to rural e-commerce, and the infrastructure construction is complete, laying the foundation for the breakthrough development of the digital countryside. With the support of "One Belt, One Road" and related national policies, the western region is gradually developing digital village-related industries. To this end, the following countermeasures are proposed.

First, explore differentiated regional digital economy development strategies to bridge the regional digital divide.

Grasp the differences between the countryside and urban areas, combine them into the spatial and temporal distribution of the constraint index of the coupling and coordination degree of China’s digital economy and rural revitalization system, and adopt a regionally differentiated digital economy investment strategy. Moderately condition the financial investment in digitalization over-saturated with digital economy development and transfer it to areas where digital economy development seriously lags behind the digitalization needs of rural revitalization. The adjustment can break through the digitalization bottleneck constraints soon as possible. Expand the digital infrastructure reach boundary in the backward areas of central and western China, continuously increase investment in digital infrastructure construction, improve network coverage, provide remote areas with stable Internet access, and focus on upgrading hardware facilities to bridge the "access gap" in backward areas. In addition, we will coordinate the construction and use of information resources in urban and rural areas, open up the existing compartmentalized information systems related to agriculture, promote the sharing and opening up of big data on the whole industrial chain of critical agricultural products, ensure the effective integration of essential data resources in agriculture and rural areas, and bridge the gap between urban and rural data facilities. Targeted financial support will be provided to people and enterprises with a low level of digitalization, such as farmers, the elderly, and small and medium enterprises, to expand the application scenarios of the digital economy and improve the universality and sharing of the digital economy. Digitalization in remote areas, in particular, needs to be strengthened so that the masses can actually enjoy the convenience brought about by digitalization, increase their work efficiency, improve their quality of life, and effectively enhance their sense of access and well-being.

Secondly, we should make extensive use of regional advantages and enhance the coordination effect of regional development. From the analysis of the spatial autocorrelation of the coupling and coordination degree of China’s digital economy and rural revitalization, it is found that the coupling and coordination degree between regions has significant spatial clustering and positive spatial correlation. This spatial linkage effect should be fully utilized, and for the eastern region with high—high concentration, it should continue to strengthen and improve its radiation effect on neighboring provinces, broaden the radiation range, enhance its interconnection and interaction with neighboring provinces, and drive the development of digital economy and rural revitalization in more neighboring provinces.

Thirdly, building a growth pole in the central and western regions. The development level of the digital economy and rural revitalization in the central and western regions is relatively low compared with the eastern region. According to the findings of the previous analysis, the Central and Western Development Center can be built around representative regions such as Xinjiang, Sichuan, and Heilongjiang to strengthen regional spatial linkage and lead the coupled development of digital economy and rural revitalization in the Central, Western, and Northeastern regions.

Fourth, we need to improve the "nurturing, attracting, and retaining talents in the digital economy" mechanism in the backward villages. This will help close the gap between regions.

The main source of the overall difference in the coupling and coordination between China’s digital economy and rural revitalization is the inter-regional difference. To narrow the inter-regional gap, the main strategic roles of digital talents and rural residents should be brought into play. We should rely on the cultivation project of new business entities to achieve "precise talent cultivation" and vigorously cultivate highly qualified agricultural talents with digital skills to bridge the "capability gap" of farmers. Furthermore, the government should strengthen training and support work between government organizations and agricultural colleges, enterprises, and institutions, as well as provide policy support in terms of treatment and development opportunities for talents, in order to retain them for a long time and improve human capital for rural revitalization in backward areas.
